# Evaluating the effect of digital technology on the learning of orthodontic cephalometric measurement

**DOI:** 10.12688/f1000research.109876.2

**Published:** 2022-12-12

**Authors:** Xin Yu, Yu Tian, Dandan Li, Wen Sun, Hua Wang, Siyang Yuan, Bin Yan, Lin Wang, Yongchu Pan

**Affiliations:** 1 Department of Orthodontics, Affiliated Nantong Stomatological Hospital of Nantong University, Nantong, 226006, China; 2 Department of Orthodontics, The Affiliated Stomatological Hospital of Nanjing Medical University, Nanjing, 210029, China; 3 Jiangsu Key Laboratory of Oral Diseases, Jiangsu Key Laboratory of Oral Diseases, Nanjing, 210029, China; 4 School of Dentistry, University of Dundee, Dundee, DD1 4HN, UK

**Keywords:** orthodontic teaching; cephalometric measurement; traditional hand-drawn method; digital technology; the Dolphin software

## Abstract

**Background:** This study aimed to evaluate the effectiveness of integrating digital technology into cephalometric measurement teaching.

**Methods:** In total, 94 undergraduates of stomatology were recruited and randomly allocated to two groups. According to the cross-over design, both groups completed cephalometric measurements by the traditional hand-drawn method and digital technology (the Dolphin software) in different order. In the traditional hand-drawn method, students depicted the outline of the craniofacial anatomical structures on sulfuric transfer paper first, then marked the measurement points and completed the measurement of line spacings and angles; by digital technology, they marked the points in the software and adjust the automatically generated outlines of the structures to obtain the results. Two professional orthodontists were invited as instructors and their measurements were set as standards. An online questionnaire was also used to investigate students' attitudes toward digital technology being used in the cephalometric teaching process.

**Results:** There were significant differences of students’ measurements ( *P*_1-SNA_<0.01, *P*_1-SNB_=0.01 and *P*_1-L1-NB (mm)_<0.01; SNA: sella-nasion-subspinale angle, SNB: sella-nasion-supramental angle, L1-NB (mm): the distance from the lower central incisor tip to the nasion-supramental plane) between the traditional method and digital technology. Besides, the results of most items by digital technology were closer to the standards than those by the traditional method, including five items with statistical significance ( *P*_2-SNB_<0.05, *P*_2-L1-NB (mm)_<0.01, *P*_2-FMA_<0.05, *P*_2-FMIA_<0.05, *P*_2-IMPA_<0.01), while three items were the opposite ( *P*_2-SNA_<0.05, *P*_2-ANB (mm)_<0.01, *P*_2-NA-PA_<0.01). The questionnaire showed more students preferred digital technology (33%) as a better teaching method than the traditional method (2%) and 72% of participants thought they had mastered 50-80% of cephalometric knowledge after the course.

**Conclusions:** This study demonstrated effectiveness and acceptance of the course applying digital technology during the cephalometric teaching process.

## Introduction

Orthodontics, as a discipline of stomatology, aims to study various kinds of malocclusion, including deformities of teeth, jaws and the craniofacial region. ^1^ Diagnosis is the most important part of orthodontic clinical work, among which cephalometric measurement is an essential procedure. ^2^ Nevertheless, cephalometric measurement indicators are numerous and complicated. In the past, the traditional hand-drawn method was applied, which required reading lamps, sulfuric acid transfer paper, dividers and so on. In addition, previous studies suggested that undergraduates tended to show less confidence in reading and measuring lateral cranial radiographs. ^3^

As continuous developments are seen in digital technology, such technology has been applied in the education for undergraduates in recent decades. Digital technology possesses vivid images and operable processes, making it more intuitive, interactive and understandable than simply imparting theoretical knowledge to students, which may help improve the effect of teaching practice. ^4^^–^^6^ Buchanan JA *et al*. found that before starting clinical work, students could better master theoretical knowledge through simulated operation or computer-aided learning method. ^7^^,^^8^ It was also reported that students' attitudes towards computer-aided learning and digital technique were positive. ^9^^–^^13^ Digital technology has offered great potential for dental education as well. ^14^ For instance, Nagy ZA *et al*. reported that the Dental Teacher software could help students more efficiently learn the preparation technique of onlay restorations and facilitate their individual performances. ^15^ Liu L *et al*. also found the digital training system might be a good alternative to the traditional training method in the preclinical practice of tooth preparation. ^16^

The digital cephalometric analysis system, widely incorporated in intelligent software, was developed to computerize the manual tasks and output the specific results automatically. It was reported to be more time-saving than traditional measurement method and helpful for reducing unnecessary errors during the measurement process. ^17^ Farooq *et al.* also found that the accuracy of cephalometric measurement by digital tracing with FACAD ^®^ was similar with the manual method. Furthermore, its advantages of digital imaging, such as quality improvement, file transmission and archiving made digitalized cephalometric analysis preferrable in daily use. ^18^

The Dolphin software ^®^ (Dolphin Imaging & management solution, America) is widely applied in the field of orthodontics, possessing functions like storage and management of patients’ information and images. After users upload computerized tomography photographs, it can also achieve three-dimensional imaging, cephalometric measurement and treatment effect prediction. It incorporates more than 400 cephalometric analytical methods. This software has been reported to have the potential as an animation textbook for medical college students. ^19^ Although it was assumed to exert some positive effects in the teaching process, there is still lack of research investigating the effectiveness of applying it in orthodontic cephalometric teaching process. Thus, the aim of this study was to examine the effectiveness of applying digital technology (the Dolphin software ^®^) in teaching cephalometric measurement.

## Methods

### Ethical considerations

This study followed the guidelines of the Nanjing Medical University ethics review committee and received the approval of the committee (approval number: PJ2019-053-001). All participants gave written informed consent.

### Study size

G*Power software version 3.1.9.7 (RRID:SCR_013726) was used to estimate the required sample size for this study. This study used two independent *t* tests to calculate the number of students needed. The study power was set at 90% and alpha value set at 0.05. Based on these, a minimum sample of 86 subjects was required.

### Participants

This study approached fourth-year undergraduate students of Stomatology in Nanjing Medical University by inviting them to attend this course, followed by their voluntary registration. All the students, consisting of 63 female and 31 male students (around 21-23 years old), agreed to participate in this pedagogical experiment and signed the informed consent form. All the students did not learn about the method of measuring the lateral cranial radiographs and use the Dolphin software before. We also invited two orthodontist faculty members with over 5 years of clinical experience as instructors.

### Study design

**Figure 1.**
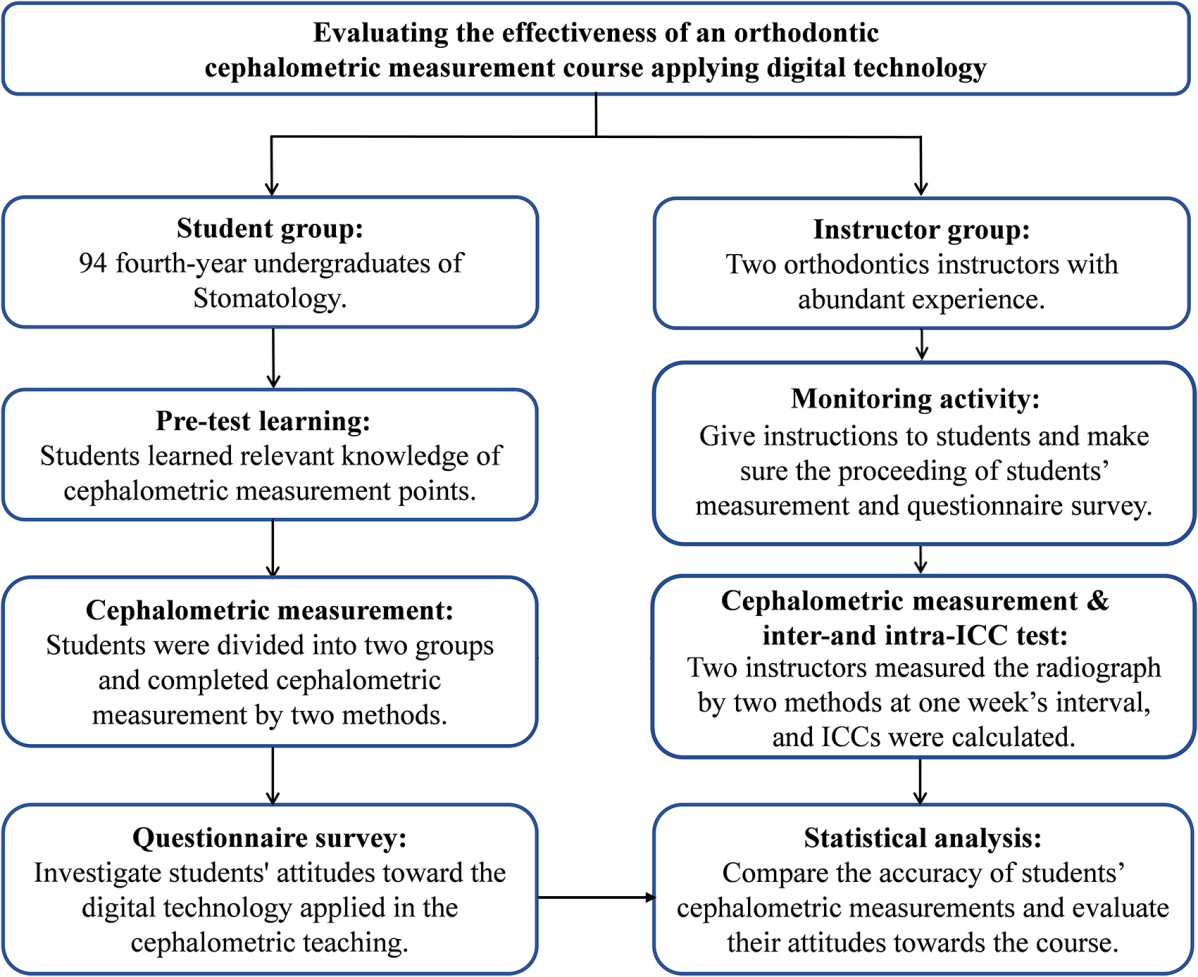
Flow diagram of the teaching experiment.

This study was conducted from May 12, 2020 to June 16, 2020. The flow diagram of this pedagogical study was shown in Figure 1. For students, it was divided into pre-test learning, group and cephalometric measurement, and questionnaire survey. For instructors, it was divided into monitoring activity, cephalometric measurement and inter- and intra-class correlation coefficient of their measurements.

***Part of students***

*Pre-test learning*

The total teaching period consisted of 7 credit hours, including 4 credit hours of theoretical class and 3 credit hours for practical instruction. During the theoretical class, one instructor imparted relevant knowledge to the students in detail, including the positions of anatomical markers and the meanings of commonly used measurement items.

In the practical instruction class, another instructor guided 94 students to review the basic knowledge and showed them how to complete cephalometric measurement by the traditional method and digital technology (the Dolphin software ^®^). For the traditional method, the sulfuric acid transfer paper was fixed to the radiograph with a clip. Then, the patient’s soft tissue profile and hard tissue anatomical structures were depicted on the reading lamp. Finally, the commonly used anatomical points were identified on the sulfuric acid transfer paper and the measurement was completed with the ruler and protractor.

While using digital technology, the instructor adjusted results of line spacing on the lateral cranial radiograph to their actual size at first. Then, the instructor accomplished the measurement by adjusting the gray contrast value and other auxiliary methods. After learning the relevant knowledge of commonly used cephalometric measurement points and items, students were encouraged to review relevant contents after class.

*Group and cephalometric measurement*

One week after the end of the pre-test instruction, 94 students were randomly allocated into two groups through the RAND function in Excel software (Microsoft ^®^ Excel 2019MSO (2201 Build 16.0.14827.20198 version for 64 bit) and were required to complete the same cephalometric measurement by both traditional method and digital technology (the Dolphin Imaging ^®^ 11.8).

**Figure 2.**
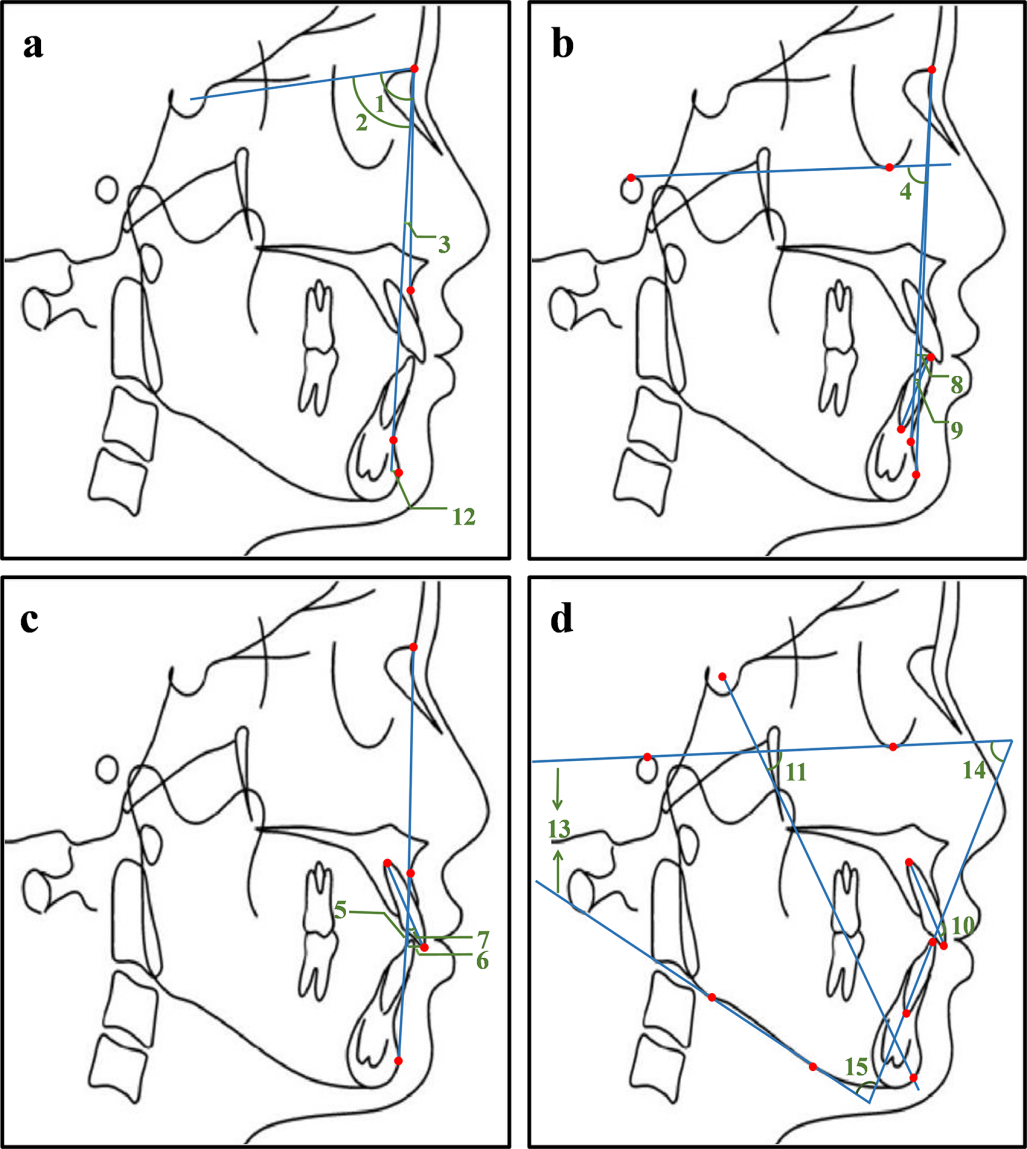
Illustration of 15 commonly used measurement items. 1. SNA: the angle between the sella-nasion plane and the nasion-subspinale plane; 2. SNB: the angle between sella-nasion plane and the nasion-supramental plane; 3. ANB: the angle between the nasion-subspinale plane and the nasion-supramental plane; 4. NP-FH: the posterior-inferior angle between the facial plane and the Frankfort horizontal plane; 5. NA-PA: the angle between the nasion-subspinale plane and the pogonion-subspinale plane; 6. U1-NA (mm): the distance from the upper central incisor tip to the nasion-subspinale plane; 7. U1-NA: the angle between the upper central incisor and the nasion-subspinale plane; 8. L1-NB (mm): the distance from the lower central incisor tip to the nasion-supramental plane; 9. L1-NB: the angle between the lower central incisor and the nasion-supramental plane; 10. U1-L1: the angle between the upper central incisor and the lower central incisor; 11.Y axis angle: the anterior-inferior angle between the Frankfort horizontal plane and the sella-pogonion plane; 12. Po-NB (mm): the distance from the pogonion point to the nasion-supramental plane; 13. FMA: the angle between the Frankfort horizontal plane and the mandibular plane; 14. FMIA: the angle between the Frankfort horizontal plane and the long axis of the lower central incisor; 15. IMPA: the angle between the long axis of the lower central incisor and the mandibular plane. The profile tracing was completed with the Uceph software (Uceph, Chengdu, China).

According to the cross-over design, one group took the traditional method first to complete the measurement and then used the Dolphin software ^®^, while the other group completed in the opposite order. A total of 15 cephalometric items ( Figure 2) were measured, such as the angle between Sella-Nasion plane and the Nasion-Subspinale plane (SNA), the angle between Sella-Nasion plane and the Nasion-Supramental plane (SNB), the angle between the Nasion-Subspinale plane and the Nasion-Supramental plane (ANB). The measurements of the traditional method and Dolphin software ^®^ were recorded, respectively. The collection and input of these data were completed by three postgraduates, with two postgraduates responsible for the inputting and the third one in charge of checking.

### Questionnaire survey

In order to survey the effectiveness of applying the digital software in cephalometric teaching and students’ attitudes toward it (the Dolphin software ^®^), we designed an online questionnaire and collected results by Wenjuanxing (Ranxing Co. Ltd., Changsha, China). For example, to investigate how difficult students considered cephalometry is, we set three options ranging from “very difficult”, “kind of tough” to “easy”. As to the mastery degree of students after the course, the options were “50-80%”, “20-50%” to “0-20%”. Gender of the participants was recorded from the university records. The detailed questionnaire list and corresponding options are shown in Table 1.

**Table 1.** Students' attitudes towards the application of digital technology.

Questionnaire list	Option list		
Are you interested in studying cephalometric measurement?	A: very interested (55%)	B: a little interested (43%)	C: not interested at all (2%)
How helpful do you think cephalometric measurement is to the diagnosis of malocclusion?	A: very helpful (77%)	B: a little helpful (21%)	C: not helpful at all (2%)
How tough do you think cephalometric measurement is?	A: very difficult (21%)	B: kind of tough (66%)	C: easy (13%)
Which do you prefer as the better teaching method?	A: digital technology (33%)	B: traditional method (2%)	C: both are acceptable (65%)
How well do you think you master the cephalometric measurement analysis?	A: 50%-80% (72%)	B: 20%-50% (28%)	C: 0-20% (0%)

***Part of instructors***

*Monitoring activity*

During this pedagogical experiments, two instructors took responsibility for guiding students with cephalometric measurements, assuring the measurement order of two student groups, monitoring the process of giving out and recollecting the questionnaire, as well as assistance in the data analysis.

*Cephalometric measurement & Inter- and intra-class correlation coefficient test*

Two instructors also measured the same radiograph by two methods. One week later, they measured the same radiograph by two methods again. The measurements by one instructor at one-week’s interval were used for calculating intra-class correlation coefficient (ICC _intra_), and measurements by two instructors were used for calculating inter-class correlation coefficient (ICC _inter_).

### Statistical analysis

The quantitative data of cephalometric measurements were analyzed by the statistical software SPSS 18.0 (IBM Corporation, Armonk, NY, RRID:SCR_016479). The measurements of students by two methods were compared using the independent sample *t* test, as well as comparing them with corresponding standards, respectively, with the level of significance set as *P*<0.05.

As for questionnaire data, we obtained the statistical data through the built-in function on the online questionnaire platform, as it provided the constituent ratio of each option and participants list. Then we performed a descriptive analysis of these results.

*Anatomical points calibration*

Before the course, the standard of anatomical markers on lateral cranial radiographs were calibrated. Firstly, two orthodontists reviewed the basic definitions and meanings of common anatomical points and items. Then, they measured a lateral cranial radiograph on paper and by software, followed by discussing and unifying the anatomical position standard. Subsequently, they respectively completed cephalometric measurement of another lateral cranial radiograph. The intraclass correlation coefficient (ICC) was greater than 90%, showing consistency of cephalometric measurement between them.

## Results

### Standards of cephalometric measurement

As shown in **Supplementary Table 1 and 2**, the inter- and intra-ICC were all surpassed 90%, and there were no significant differences between two instructors’ measurements, and the measurements by two methods. As the result, the means of two instructors’ measurements were set as the standards.

### Accuracy of students’ cephalometric measurement

Statistically significant differences were observed in measurements of SNA, SNB and the distance from the lower central incisor tip to the nasion-supramental plane (L1-NB (mm)) between the traditional method and digital technology ( *P*_1-SNA_<0.01, *P*_1-SNB_=0.01, *P*_1-L1-NB (mm)_<0.01) ( Table 2), while other items showed no significant differences. Besides, the measurements by digital technology were closer to the standard values than those by the traditional method. The accuracy of five items measurements using digital technology was significantly higher, including SNB, L1-NB (mm), the angle between the Frankfort horizontal plane and the mandibular plane (FMA), the angle between the Frankfort horizontal plane and the long axis of the lower central incisor (FMIA), and the angle between the long axis of the lower central incisor and the mandibular plane (IMPA) ( *P*_2-SNB_<0.05, *P*_2-L1-NB (mm)_<0.01, *P*_2-FMA_<0.05, *P*_2-FMIA_<0.05, *P*_2-IMPA_<0.01). However, five items presented the opposite result, among which three items were statistically significant (SNA, ANB and the angle between the Nasion-Subspinale plane and the pogonion-subspinale plane (NA-PA) ( *P*_2-SNA_<0.05, *P*_2-ANB_<0.01, *P*_2-NA-PA_<0.01).

**Table 2.** Comparison of students' measurements and standard values by the digital technology and traditional method.

Measurement items	Digital technology			Traditional method			*P*_1_	*P*_2_
	Test value ( $\bar{X}$ ±SD)	Standard value	${\bar{d}}_{1}$	Test value ( $\bar{X}$ ±SD)	Standard value	${\bar{d}}_{2}$		
SNA (°)	92.46±1.88	92.00	1.53	91.41±1.47	92.10	1.19	**<0.01**	**<0.05**
SNB (°)	88.47±0.86	88.30	0.69	88.03±1.35	88.30	0.94	**0.01**	**<0.05**
ANB (°)	3.76±1.83	3.70	1.43	3.50±0.86	3.80	0.75	0.25	**<0.01**
NP-FH (°)	89.32±1.62	90.70	1.83	88.95±1.61	90.90	1.89	0.13	0.76
NA-PA (°)	5.99±3.60	6.40	2.79	5.98±1.86	6.50	1.56	0.98	**<0.01**
U1-NA (mm)	5.07±1.86	6.20	1.71	5.20±1.41	6.30	1.35	0.61	0.06
U1-NA (°)	21.26±3.77	19.70	3.41	21.69±4.75	19.90	3.88	0.50	0.26
L1-NB (mm)	6.09±0.48	6.70	0.73	5.76±0.75	6.80	1.00	**<0.01**	**<0.01**
L1-NB (°)	25.10±2.24	26.45	2.19	25.69±3.40	26.60	2.68	0.16	0.09
U1-L1 (°)	129.68±4.67	130.25	4.04	130.16±5.97	130.50	4.82	0.54	0.08
Y axis (°)	64.45±0.99	62.80	1.79	64.22±1.93	62.70	1.91	0.32	0.52
Po-NB (mm)	1.13±0.46	1.05	0.37	1.21±0.44	1.02	0.35	0.21	0.54
FMA (°)	28.89±2.05	28.25	1.71	29.31±2.51	28.50	2.16	0.22	**<0.05**
FMIA (°)	63.46±2.78	63.75	2.27	63.36±3.52	63.80	2.90	0.83	**<0.05**
IMPA (°)	87.94±2.78	88.00	2.31	87.80±4.03	88.20	3.30	0.79	**<0.01**

X: Mean of students’ measurements; SD: Standard deviation of students’ measurements; Standard value: Mean of two orthodontists’ measurements by traditional method or digital technology; ${\bar{d}}_{1}$ : Mean of absolute values of differences between students’ results and standard values by digital software; ${\bar{d}}_{2}$ : Mean of absolute values of differences between students’ results and standard values by traditional method; *P*_1_: The *P* value of the independent sample *t* test on students’ measurements by two methods; *P*_2_: The *P* value of the independent sample *t* test on absolute values of differences between students’ results and the standards by two methods.

SNA: the angle between the sella-nasion plane and the nasion-subspinale plane; SNB: the angle between sella-nasion plane and the nasion-supramental plane; ANB: the angle between the nasion-subspinale plane and the nasion-supramental plane; NP-FH: the posterior-inferior angle between the facial plane and the Frankfort horizontal plane; NA-PA: the angle between the nasion-subspinale plane and the pogonion-subspinale plane; U1-NA (mm): the distance from the upper central incisor tip to the nasion-subspinale plane; U1-NA: the angle between the upper central incisor and the nasion-subspinale plane; L1-NB (mm): the distance from the lower central incisor tip to the nasion-supramental plane; L1-NB: the angle between the lower central incisor and the nasion-supramental plane; U1-L1: the angle between the upper central incisor and the lower central incisor; Y axis angle: the anterior-inferior angle between the Frankfort horizontal plane and the sella-pogonion plane; Po-NB (mm): the distance from the pogonion point to the nasion-supramental plane; FMA: the angle between the Frankfort horizontal plane and the mandibular plane; FMIA: the angle between the Frankfort horizontal plane and the long axis of the lower central incisor; IMPA: the angle between the long axis of the lower central incisor and the mandibular plane.

### Attitudes of students towards the digital technology

We assigned the questionnaires to all the participants with 82 of 94 students filling out the questionnaire and the response rate was 87%. The statistical results of the questionnaire are shown in Table 1. Among the respondents, 66% thought studying cephalometry was very difficult and 21% thought it was kind of tough. After instruction, review and practice, 72% of them considered they had mastered 50-80% of relevant knowledge and a few students thought they had mastered 20-50%. About 33% of students preferred the digital technology than traditional method (2%) as a better teaching method and 65% held that both were acceptable, which indicated good acceptance by students of digital technology applied in the teaching process. In addition, 98% of participants expressed their interest in studying cephalometry and considered cephalometric analysis helpful to diagnosis of malocclusion.

## Discussion

Cephalometric measurement is essential for diagnosis and treatment plan design of patients with malocclusions, which are the most significant procedures in orthodontic clinical work. Orthodontic educators put forward that orthodontic teaching for undergraduates should focus on diagnosis and recognition of problems. ^20^ However, there is not a generally accepted teaching method, and a wide variation of course durations and contents exist in different dental colleges and faculties. ^21^ How to arouse students’ interest and achieve better teaching effects is a major problem faced by orthodontic educators. Since only a few reports explored this aspect, we designed this didactical experiment.

In our study, three cephalometric items measured by two methods were statistically different, while other items were basically similar. These results implied that digital technology could achieve similar results to the traditional method during cephalometric measurement. Additionally, the majority of items measured by digital technology were closer to the standards, including five statistically significant items, which suggested students could achieve more accurate results by digital technology. This may be attributed to the function of digital software to adjust the gray contrast value of X-ray films (Supplementary figure 1), making it easier to identify the unclear points on the printed paper. In addition, the automatic generation of results also helped to avoid evitable errors during manual measurement. These results were in accordance with previous studies, which suggested that the accuracy of digital measurement on 3-dimensional cone beam computed tomography images was basically similar to or even higher than that of manual measurement. ^22^^,^^23^

However, in spite of the convenience it provided, the digital technology may lead to a lack of deep understanding of corresponding contents, such as definitions and meanings of these measurement items. The traditional method could better cultivate practical abilities of students and enhance their memory of relevant knowledge. Besides, although the accuracy of some items obtained by digital technology were significantly higher, a few items showed the opposite result (SNA, ANB and NA-PA). The subspinale point (the A point) was associated with these three items, which suggested that the traditional method was more accurate than digital technology in positioning the subspinale point on the lateral cranial radiograph. The subspinale point is the most concave point of the arc from the anterior nasal spine point (the ANS point) to the superior prosthion point (the Spr point). Compared with digital measurement, the advantage of manual measurement is that the arc can be traced on paper, and some auxiliary instruments like ruler and protractor can help to locate the subspinale point (Supplementary figure 2). These results indicated abundant experience was required to identify the subspinale point, reminding both orthodontic educators and students to devote more time and energy to deep learning it.

Before the course, the majority of students showed fear towards abstract and complex concepts, assuming cephalometric measurement was difficult to master. However, with instruction and practice, most students could master 50-80% of relevant knowledge, which could result from digital technology realizing visualization of numerous and complicated anatomical markers. Previous studies found that visualization was extremely attractive to young students, and significantly aroused their interest and sense of participation. ^24^^,^^25^ Our survey confirmed that more students preferred digital technology (33%) as a better teaching method than the traditional method. As a result, the application of digital technology in teaching cephalometric measurement was widely accepted by students and contributed to favorable teaching results.

This study still had some limitations. For example, the sample size could be further enlarged. Secondly, the measurement time for students were not strictly required, which may have resulted in the underperformances or supernormal performances of students. Thirdly, exploration of student’s attitudes towards digital technology being applied in this course was not sufficient. The mentioned issues needed to be improved may interrupt us from to accurately assessing the real advantages and disadvantages of the appliance of this digital technology. These findings could guide and encourage university orthodontic teachers to apply this technology in cephalometric teaching and pay more attention to considering the position identification of the subspinale point.

## Conclusion

This study investigated the effect of applying the digital technology (the Dolphin software ^®^) in cephalometric learning, demonstrating better effect and acceptance of this technology.

## Data Availability

Figshare: Evaluating the effect of digital technology on the learning of orthodontic cephalometric measurement, https://doi.org/10.6084/m9.figshare.21550599.v1. ^26^ This project contains the following extended data:
- Supplementary File.docx (Supplementary figure 1, 2; Supplementary table 1, 2) - The results of students’ measurements.xlsx - Statistical results of the questionnaire.xlsx Data are available under the terms of the Creative Commons Attribution 4.0 International license (CC-BY 4.0).
