# Supplementary material for: Evaluating the effect of digital technology on the learning of orthodontic cephalometric measurement
Source: F1000Res. 2023 Jun 13;11:328. Originally published 2022 Mar 18. [Version 3] doi: 10.12688/f1000research.109876.3 (PMC10674087; doi:10.12688/f1000research.109876.3)
Supplement: Peer review history

# Reviewer report: Reviewer response for version 3

**DOI:** 10.5256/f1000research.144525.r178260

**Published:** 2023-11-24

**Authors:** Ana Corte-Real

**Affiliations:** 1 Faculty of Medicine, University of Coimbra, Coimbra, Portugal; 2 Forensic Dentistry Laboratory, Faculty of Medicine, University of Coimbra, Coimbra, Portugal

The authors correspond positively to the suggestions.

Is the work clearly and accurately presented and does it cite the current literature?

No

If applicable, is the statistical analysis and its interpretation appropriate?

No

Are all the source data underlying the results available to ensure full reproducibility?

No

Is the study design appropriate and is the work technically sound?

No

Are the conclusions drawn adequately supported by the results?

No

Are sufficient details of methods and analysis provided to allow replication by others?

No

Reviewer Expertise:

NA

I confirm that I have read this submission and believe that I have an appropriate level of expertise to confirm that it is of an acceptable scientific standard.

# Reviewer report: Reviewer response for version 2

**DOI:** 10.5256/f1000research.140872.r157996

**Published:** 2023-01-11

**Authors:** Ana Corte-Real

**Affiliations:** 1 Faculty of Medicine, University of Coimbra, Coimbra, Portugal; 2 Forensic Dentistry Laboratory, Faculty of Medicine, University of Coimbra, Coimbra, Portugal

The authors improved the manuscript. However, some suggestions should be pointed out in the Methods section.

- "All the students use (/used?) the Dolphin software before"… is it really what the authors intended to write?
- The original information of the manuscript is its impact on orthodontic learning and students' new skills and satisfaction (instead of "student's acceptance towards it"). It is difficult to understand the sentence, "The collection and input of these data were completed by three postgraduates, with two postgraduates responsible for the inputting and the third one in charge of checking." It should be clarified. The student's contribution to the cephalometric analysis and their performance and satisfaction evaluations should be clear. 
- Intra and inter-class correlations were based on the instructors' data. What is its impact on the present study?

Is the work clearly and accurately presented and does it cite the current literature?

No

If applicable, is the statistical analysis and its interpretation appropriate?

No

Are all the source data underlying the results available to ensure full reproducibility?

No

Is the study design appropriate and is the work technically sound?

No

Are the conclusions drawn adequately supported by the results?

No

Are sufficient details of methods and analysis provided to allow replication by others?

No

Reviewer Expertise:

Medical and Dentistry Higher Education and Health Quality

I confirm that I have read this submission and believe that I have an appropriate level of expertise to confirm that it is of an acceptable scientific standard, however I have significant reservations, as outlined above.

# Response

**Published:** 2023-02-20

**Authors:** Yu Tian

**Affiliations:** Jiangsu Key Laboratory of Oral Diseases, China

**The authors improved the manuscript. However, some suggestions should be pointed out in the Methods section.**

**Response: **We are grateful for the reviewer’s helpful suggestions. As shown below, we have addressed the reviewer’s concerns, and provided point-by-point responses. We hope that the changes meet your approval.

**1. "All the students use (/used?) the Dolphin software before"… is it really what the authors intended to write?**

**Response: **Thank you for pointing out this mistake. We have revised the sentence to “All the students have neither learned about the method of measuring the lateral cranial radiographs nor used the Dolphin software before.”

**2. The original information of the manuscript is its impact on orthodontic learning and students' new skills and satisfaction (instead of "student's acceptance towards it").**

**Response: **Thank you for your suggestion. According to your advice, we have revised the phrase to “orthodontic learning, students' new skills and satisfaction towards it”.

**3. It is difficult to understand the sentence, "The collection and input of these data were completed by three postgraduates, with two postgraduates responsible for the inputting and the third one in charge of checking." It should be clarified. The student's contribution to the cephalometric analysis and their performance and satisfaction evaluations should be clear.**

**Response: **Thank you for your suggestion. To make it clear, we have revised the sentence to “The collection, input and check of these data were completed by three authors.”

**4. Intra and inter-class correlations were based on the instructors' data. What is its impact on the present study?**

**Response: **Thank you for your question. In order to evaluate students’ performances, we needed standards. Thus, we invited the experienced instructors and set their measurement results as standards after checking the stability and consistency of their measurements.

# Reviewer report: Reviewer response for version 2

**DOI:** 10.5256/f1000research.140872.r157997

**Published:** 2023-01-04

**Authors:** Cinzia Maspero

**Affiliations:** 1 Fondazione IRCCS Cà Granda, Ospedale Maggiore Policlinico, Milan, Italy; 2 Department of Orthodontics, Faculty of Medicine, University of Milan, Milan, Italy

All my requirements have been addressed. The manuscript has been improved and, in my opinion, it is suitable for indexing.

Is the work clearly and accurately presented and does it cite the current literature?

Yes

If applicable, is the statistical analysis and its interpretation appropriate?

Yes

Are all the source data underlying the results available to ensure full reproducibility?

Yes

Is the study design appropriate and is the work technically sound?

Partly

Are the conclusions drawn adequately supported by the results?

Partly

Are sufficient details of methods and analysis provided to allow replication by others?

Yes

Reviewer Expertise:

Orthodontic, Orthognati surgery, cephalometic, maxillary expansion, interceptive orthodontics, fixed appliances, clear aligners, pediatric dentistry

I confirm that I have read this submission and believe that I have an appropriate level of expertise to confirm that it is of an acceptable scientific standard.

# Reviewer report: Reviewer response for version 1

**DOI:** 10.5256/f1000research.121428.r135780

**Published:** 2022-09-07

**Authors:** Ana Corte-Real

**Affiliations:** 1 Faculty of Medicine, University of Coimbra, Coimbra, Portugal; 2 Forensic Dentistry Laboratory, Faculty of Medicine, University of Coimbra, Coimbra, Portugal

The topic is interesting for assessing learning from the student's perspective, but the title does not correspond to the content. The methodology section is the weakest part of the work. The authors should adjust the study design, including the analysis of intra and inter-observer accuracy and monitoring activities during the study. The selection of participants should include the inclusion and exclusion criteria as the previous use of digital technology for cephalometric measurements impacts the students' skills.

Is the work clearly and accurately presented and does it cite the current literature?

No

If applicable, is the statistical analysis and its interpretation appropriate?

No

Are all the source data underlying the results available to ensure full reproducibility?

No

Is the study design appropriate and is the work technically sound?

No

Are the conclusions drawn adequately supported by the results?

No

Are sufficient details of methods and analysis provided to allow replication by others?

No

Reviewer Expertise:

Medical and Dentistry Higher Education and Health Quality

I confirm that I have read this submission and believe that I have an appropriate level of expertise to state that I do not consider it to be of an acceptable scientific standard, for reasons outlined above.

# Response

**Published:** 2022-11-07

**Authors:** Yu Tian

**Affiliations:** Jiangsu Key Laboratory of Oral Diseases, China

**Response: **We are grateful for the reviewer’s helpful suggestions. As shown below, we have addressed the reviewer’s concerns, and provided point-by-point responses. We hope that the changes meet your approval.

**1. The topic is interesting for assessing learning from the student's perspective, but the title does not correspond to the content.**

**Response: **Thank you for your valuable suggestions. We fully understand and agree with your suggestion about the relationship between the title and content of this article. We’ve revised the title to make it more suitable for this study “Evaluating the effect of digital technology on the learning of orthodontic cephalometric measurement”.

**2. The methodology section is the weakest part of the work. The authors should adjust the study design, including the analysis of intra and inter-observer accuracy and monitoring activities during the study.**

**Response: **Thank you for your valuable suggestion. We added in **Methods **section that “The measurements by one instructor at one-week’s interval were used for calculating intra-class correlation coefficient (ICC _intra_), and measurements by two instructors were used for calculating inter-class correlation coefficient (ICC _inter_).”, and in **Standards of cephalometric measurement **section that “The inter- and intra-ICC were all surpassed 90%, and there were no significant differences between two instructors’ measurements, and the measurements by two methods”.

 As regard to the monitoring activity, we’ve added that “During this pedagogical experiments, two instructors took responsibility for guiding students with cephalometric measurements, assuring the measurement order of two student groups, monitoring the process of giving out and recollecting the questionnaire, as well as assistance in the data analysis.” in the ***Monitoring activity*** section in page 8.

**3. The selection of participants should include the inclusion and exclusion criteria as the previous use of digital technology for cephalometric measurements impacts the students' skills.**

**Response: **Thank you for your suggestions. We agree with your advice on the selection of participant students. We’ve added in “Participants” section in line 5, page 6 that “…All the students did not learn about the method of measuring the lateral cranial radiographs and used the Dolphin software before”.

# Reviewer report: Reviewer response for version 1

**DOI:** 10.5256/f1000research.121428.r148740

**Published:** 2022-09-07

**Authors:** Cinzia Maspero

**Affiliations:** 1 Fondazione IRCCS Cà Granda, Ospedale Maggiore Policlinico, Milan, Italy; 2 Department of Orthodontics, Faculty of Medicine, University of Milan, Milan, Italy

Thank you for submitting your research. The manuscript is interesting but some changes are necessary before taking it into consideration for publication. Here are my concerns:

- ABSTRACT: "Besides, the results of most items by digital technology were closer to the **standards** than those by the traditional method" What do you mean with standards?
- INTRODUCTION: replace the word subdiscipline with discipline.
- "These factors may not only affect accuracy of measurements, but also contribute to destruction of students’ enthusiasm for further learning." Are you sure with this sentence?
- STUDY DESIGN: pre-test instruction. Please better explain what do you mean and what instruction have been given.
- FLOW CHART: anatomical point calibration: calibrated their standard. What do you mean? Specify which standard.
- CEFALOMETRIC MEASUREMENTS: it is not clear if the same lateral teleradiography have been used for all the students. Better specify.
- Illustration of 15 commonly used measurement items: which kind of tracing have been used?
- Please better specify the aim of this study.

Is the work clearly and accurately presented and does it cite the current literature?

Yes

If applicable, is the statistical analysis and its interpretation appropriate?

Yes

Are all the source data underlying the results available to ensure full reproducibility?

Yes

Is the study design appropriate and is the work technically sound?

Partly

Are the conclusions drawn adequately supported by the results?

Partly

Are sufficient details of methods and analysis provided to allow replication by others?

Yes

Reviewer Expertise:

Orthodontic, Orthognati surgery, cephalometic, maxillary expansion, interceptive orthodontics, fixed appliances, clear aligners, pediatric dentistry

I confirm that I have read this submission and believe that I have an appropriate level of expertise to confirm that it is of an acceptable scientific standard, however I have significant reservations, as outlined above.

# Response

**Published:** 2022-11-07

**Authors:** Yu Tian

**Affiliations:** Jiangsu Key Laboratory of Oral Diseases, China

Thank you for submitting your research. The manuscript is interesting but some changes are necessary before taking it into consideration for publication. Here are my concerns:

**Response: **We are grateful for the reviewer’s helpful suggestions. As shown below, we have addressed the reviewer’s concerns, and provided point-by-point responses. We hope that the changes meet your approval.

**1. ABSTRACT: "Besides, the results of most items by digital technology were closer to the standards than those by the traditional method" What do you mean with standards?**

**Response: **Thank you for your valuable suggestion. The means of two instructors’ measurements were set as the standards, and we’ve added this in the abstract (line 4 in Results, page 2).

**2. INTRODUCTION: replace the word subdiscipline with discipline.**

**Response: **Thank you for your suggestion. According to your advice, we’ve used the word “discipline” in line 1, page3.

**3. "These factors may not only affect accuracy of measurements, but also contribute to destruction of students’ enthusiasm for further learning." Are you sure with this sentence?**

**Response: **Thank you for your advice. We’ve deleted the sentence.

**4. STUDY DESIGN: pre-test instruction. Please better explain what do you mean and what instruction have been given.**

**Response: **Thank you for your suggestion. We’ve checked the manuscript, and described the pre-test instruction in detail in page 6, which included 7 credit hours and let students learn about the 15 cephalometric items and two measurement methods.

**5. FLOW CHART: anatomical point calibration: calibrated their standard. What do you mean? Specify which standard.**

**Response: **Thank you for your valuable suggestions. We revised the subtitle and described in detail that “Before the course, the inter- and intra-class correlation coefficient of two instructors’ measurements were tested. Firstly, two orthodontists reviewed and discussed the locations of anatomical points. Then, they measured a lateral cranial radiograph by traditional and software methods. One week later, they measured the same radiograph by two methods again. The measurements by one instructor at one-week’s interval were used for calculating intra-class correlation coefficient (ICC _intra_), and measurements by two instructors were used for calculating inter-class correlation coefficient (ICC _inter_)” in **Inter- and intra-class correlation coefficient of two instructors’ measurements **section.

**6. CEFALOMETRIC MEASUREMENTS: it is not clear if the same lateral teleradiography have been used for all the students. Better specify.**

**Response: **Thank you for your suggestion. We’ve added in line 2, page 7 that “One week after the end of the pre-test instruction, 94 students were randomly allocated into two groups through the RAND function in Excel software (Microsoft® Excel® 2019MSO (2201 Build 16.0.14827.20198 version for 64 bit) and were required to complete cephalometric measurement of the same lateral cranial radiograph by both traditional method and digital technology (the Dolphin Imaging® 11.8).”.

**7. Illustration of 15 commonly used measurement items: which kind of tracing have been used?**

**Response:** Thank you for your question. The tracing was completed with the Uceph software, and we’ve added “The profile tracing was completed with the Uceph software (Uceph, Chengdu, China).” in the illustration of **Figure 2**.

**8. Please better specify the aim of this study.**

**Response: **Thank you for your suggestion. We’ve revised in abstract and introduction section that “This study aimed to evaluate the effect of applying digital technology in cephalometric measurement teaching and students’ acceptance towards it”.
